# Online Health Information Seeking for Self and Child: An Experimental Study of Parental Symptom Search

**DOI:** 10.2196/29618

**Published:** 2022-05-09

**Authors:** Christian Kubb, Heather M Foran

**Affiliations:** 1 Health Psychology Unit Institute of Psychology Universität Klagenfurt Klagenfurt Austria

**Keywords:** online health information seeking, digital health, parents, parent-child, eHealth literacy, human computer interaction, mobile phone

## Abstract

**Background:**

Parents often search the web for health-related information for themselves or on behalf of their children, which may impact their health-related decision-making and behaviors. In particular, searching for somatic symptoms such as headaches, fever, or fatigue is common. However, little is known about how psychological and relational factors relate to the characteristics of successful symptom-related internet searches. To date, few studies have used experimental designs that connect participant subjective search evaluation with objective search behavior metrics.

**Objective:**

This study aimed to examine the features of web-based health-related search behaviors based on video-coded observational data, to investigate which psychological and relational factors are related to successful symptom search appraisal, and to examine the differences in search-related outcomes among self-seekers and by-proxy seekers.

**Methods:**

In a laboratory setting, parents living in Austria (N=46) with a child aged between 0 and 6 years were randomized to search their own (n=23, 50%) or their child’s (n=23, 50%) most recent somatic symptom on the web. Web-based activity was recorded and transcribed. Health anxiety, eHealth literacy, attitude toward web-based health information, relational variables, state of stress, participants’ search appraisals, and quantitative properties of the search session were assessed. Differences in search appraisals and search characteristics among parents who searched for themselves or their children were examined.

**Results:**

Across both groups, searches were carried out for 17 different symptom clusters. Almost all parents started with Google (44/46, 96%), and a majority used initial elaborated key phrases with >1 search keyword (38/45, 84%) and performed on average 2.95 (SD 1.83) search queries per session. Search success was negatively associated with health anxiety (*r*_s_=−0.39, *P*=.01), stress after the search (*r*_s_=−0.33, *P*=.02), and the number of search queries (*r*_s_=−0.29, *P*=.04) but was not significantly associated with eHealth literacy (*r*_s_=0.22, *P*=.13). Of note, eHealth literacy was strongly and positively correlated with satisfaction during the search (*r*_s_=0.50, *P*<.001) but did not significantly correlate with search characteristics as measured by search duration (*r*_s_=0.08, *P*=.56), number of performed search queries (*r*_s_=0.20, *P*=.17), or total clicks (*r*_s_=0.14, *P*=.32). No differences were found between parents searching for their own symptoms and parents searching for their child’s symptoms.

**Conclusions:**

This study provides exploratory findings regarding relevant dimensions of appraisals for symptom-based information seeking on the web. Consistent with previous literature, health anxiety was found to be associated with poorer search evaluation. Contrary to expectations, eHealth literacy was related neither to search success nor to search characteristics. Interestingly, we did not find significant differences between self-seekers and by-proxy seekers, suggesting similar search and evaluation patterns in our sample. Further research with larger samples is needed to identify and evaluate guidelines for enhanced web-based health information seeking among parents and the general public.

## Introduction

### Background

The entry into the digital age has fundamentally altered information behavior, particularly information retrieval and information seeking [1,2]. Internet users commonly use search engines to obtain references related to all facets of everyday life [3,4]. More than 5 billion search queries are performed daily via Google [5], indicating enormous informational needs on a global level. Information retrieved from the web influences consumer behavior and decision-making in various contexts, both on the web and offline [1,6,7]. Understanding how information is sought and used has important implications for decision-making processes across a wide array of research fields in business, education, medicine, or public health [1,6,8,9].

Most notably, online health information seeking (OHIS) is a very common information behavior among all age groups and countries. In 2020, a total of 55% of European citizens aged between 16 and 74 years searched on the web about health-related topics such as disease, injury, or nutrition at least once in the past 3 months [10]. Emerging trends of individuals looking for information on health and illness can be observed around the globe [10-12]. OHIS has a crucial impact on health decision-making [13-15] and the nature of the physician-patient relationship [16]. It also provides significant opportunities for receiving social and emotional support in health affairs [17].

Large-scale studies from Europe [18] and the United States [19] imply that between 35% and 61% of web-based information seekers conducted health-related searches not only for themselves but also on behalf of others, such as for their children, friends, or relatives. In particular, parents of young children play a special mediating role, as they decide on the extent and timing of health measures for their offspring. In a recent review by Kubb and Foran [20], the prevalence rates for parental OHIS were considerably higher than those in the general population, suggesting that parents are heavy users of web-based health information. However, seeking by proxy can threaten children’s health status if the search leads to detrimental self-treatments or a delay in seeking professional health services. In particular, misleading information and medical fake news on the internet can impede decision-making and thus are a potential threat for self-health, children’s health, and public health [21-24].

Searching for somatic symptoms is common among internet users. Approximately 1% of all Google searches are symptom-related [25], with search queries on persistent or acute symptoms, such as chest pain, headache, fever, or diarrhea [26]. According to Cartright et al [27], two phases of exploratory diagnostic search are supposed: either to investigate the relevance of symptoms (evidence-directed) or to inform about certain diseases (hypothesis-directed). In this context, parents use the web to determine whether their child needs medical consultation [28-30]. However, the quality of health websites varies greatly [31,32] and is often lacking in terms of relevant information whether a symptom requires treatment [26]. More dedicated research on parental symptom search and the underlying factors for a successful search could therefore contribute significantly to better health-related decision-making by parents.

In the past, various traits and concepts were introduced for being pivotal in the context of health-related web-based searches. A growing body of literature has examined the relation between health anxiety and OHIS [33]. Broadly defined, health anxiety encompasses illness worries and excessive fears about developing or having a serious disease [34]. A meta-analysis by McMullan et al [35] found health anxiety being moderately positively correlated with OHIS across 10 studies. Further studies examined the reciprocal relationship between health anxiety and OHIS [36] or the effect on health care use [37]. Trait health anxiety is associated with poorer outcomes during as well as after a health-related web-based search, including negative emotions [38], worsening anxiety [39], and an increased number of physician visits [33,37]. A promising construct for diminishing adverse outcomes of OHIS is eHealth literacy, introduced by Norman and Skinner [40]. The term describes the ability to search, find, understand, evaluate, and ultimately use health-relevant information on the web. Previous studies suggested that higher eHealth literacy is associated with less frustration during the information search [41], gain in empowerment [42], and better evaluating skills of web-based health information [43]. In the future, targeting eHealth literacy could become a key element for enhancing OHIS skills and related outcomes [44,45].

The assessment of appraisals during or after information-seeking behavior offers opportunities for identifying the kind of information that users find helpful. In this context, search success and search satisfaction are two central concepts within users’ information evaluation processes [46-48]. Search success refers to finding an answer to a predefined question or information regarding the search topic [49,50]. In comparison, search satisfaction relates to the emotional fulfillment during the search [48,51]. Although both concepts are strongly connected, satisfaction during the search does not guarantee a successful search or vice versa [46]. To date, neither the prerequisites nor the properties of a successful and satisfactory web-based health search have been well understood. Consequently, evidence-based recommendations for parental OHIS on effective and successful OHIS are yet missing [20].

Some methodological approaches have been applied to investigate the process of OHIS sessions in depth, including video recording, think-aloud protocols, or interviews [52-58]. Unfortunately, solely the study by Benedicta et al [56] was conducted in a sample of parents, and only two studies [55,57] focused on symptom-driven queries. In addition, previous studies may no longer reflect current search behavior, as the nature of information seeking has changed significantly in recent years. New opportunities, among others, were given with web-based symptom checkers [59], highly specialized communities on specific rare diseases [60], YouTube videos [61], or smartphone health apps [62]. Therefore, there is an urgent need for more experimental research on the health-related search process on the web itself, including the assessment of long-term traits, short-term emotional states, appraisals during and after the search, and their impact on health behavior in real life.

### Objective

The aims of this study are 3-fold: (1) to categorize and analyze the performed search queries; (2) to investigate the associations of health anxiety, eHealth literacy, and eHealth attitudes with log file data and self-reported appraisals of recorded symptom-driven search sessions; and (3) to examine differences between self-seekers and by-proxy seekers in terms of log file data and self-reported appraisals. We hypothesize that health anxiety is positively correlated with poorer outcomes (ie, unmet information needs, information overload, and need to talk to a physician), whereas eHealth literacy and favorable attitudes toward web-based health information will be positively associated with beneficial search appraisals (ie, success, satisfaction, and empowerment). In addition, we presume that OHIS by proxy is accompanied with poorer search outcomes than seeking for one’s own health (eg, higher unmet information needs, more information overload, higher need to talk to a physician, lower search appraisal, more dissatisfaction, and lower levels of empowerment).

## Methods

### Inclusion and Exclusion Criteria

Participants were required to be aged ≥18 years, be a parent of a child aged between 0 and 6 years, and have used the internet at least sometimes for health-related information seeking. We began recruitment with child age range between 0 and 3 years and extended the recruitment to kindergartens, which included children aged ≤6 years. Parents or children with chronic illnesses were excluded from participation. A maximum of 1 parent per household could participate. In cases where both parents participated, one was chosen at random to maintain a sample without dependent data at the couple level.

To measure ecological validity, participants were asked at the end of the experiment how similar the expressed search behavior was to the normal at home on a 6-point Likert scale (0 *not similar at all*; 5 *exactly what I would do at home*). Participants with a score of ≤3 were excluded from the analysis (n=6).

### Recruitment

Participants were recruited predominantly with leaflets in kindergartens, pediatrician waiting rooms, playgroups, and parent-child facilities. The local media also published our call for participation. In addition, we ran local advertising on *Instagram* and *Facebook*. Participants contacted us by phone or email to set up an appointment at the university laboratory. The experiment was advertised with an estimated duration of 1 hour. As an incentive, each participant received an expense allowance of €10 (US $11) for completing the study. The chance to win a €100 voucher was raffled among all participants. Recruitment lasted from November 2019 to March 2020 and was stopped prematurely by governmental measures to contain the spreading of SARS-CoV-2 that began in March in Austria. At the point in which recruitment stopped, there were almost no cases of SARS-CoV-2 in Carinthia.

### Participants

A total of 59 individuals participated in the experiment. Of these 59 participants, 6 (10%) were excluded because they stated that their health-related web-based search was not similar to that at home. Furthermore, 12 other participants were members of a couple, and one member of each dyad was excluded at random. Unfortunately, of the 59 participants, 1 (2%) was affected by technical issues with the recording program. This resulted in a final sample of N=46, equally balanced for both experimental groups.

Participants were mainly mothers (40/46, 87%) and had Austrian citizenship (40/46, 87%). Parents were aged between 25 and 46 years (mean 33.72, SD 4.11 years). The youngest child was on average aged 28.93 (SD 17.73) months, with the youngest being aged 2 months and the oldest being aged 6 years. Of the 46 parents, 9 (20%) had a small child with them during the experiment. In addition, of the 46 parents, 44 (96%) reported being in a relationship, ranging from 1 to 20 years (mean 9.86, SD 4.5 years). The demographic characteristics of participants are reported in Table 1.

**Table 1:** Sociodemographic characteristics of participants (N=46)^a^.

		Full sample, n (%)
**Sex**		
	Female	40 (87)
	Male	6 (13)
**Nationality**		
	Austria	40 (87)
	Germany	3 (7)
	Other	3 (7)
**Marital status**		
	Unmarried	15 (33)
	Married	30 (65)
	Divorced	1 (2)
**Relationship status**		
	Single	2 (4)
	In a relationship	44 (96)
**Number of children**		
	1	17 (37)
	2	25 (54)
	3	4 (9)
**Educational level**		
	Prefer not to say	2 (4)
	Lower secondary	1 (2)
	Apprenticeship	8 (17)
	High school	5 (11)
	Tertiary education	30 (65)

^a^Participants were on average aged 33.72 (SD 4.11) years.

### Experimental Procedure

Our experimental setup was developed based on the approach of Singh and Brown [55]. Written informed consent was obtained from all participants before the study. Subsequently, participants were asked to fill out the initial test inventory on paper. A smartwatch was worn during the experiment to measure physiological responses; however, these data are not included in this study. Participants were asked to search the web for current or recent somatic health issues related to self-symptoms or child symptoms. A between-subject design with block randomization was applied to assign parents to either the self-seeking or by-proxy seeking group. The Patient Health Questionnaire-15 (PHQ-15) was used to identify recent symptoms for the participants’ search task. An adapted version to appropriate symptoms in children was presented to parents in the respective group. Participants rated on a 6-point Likert scale the likelihood that each experienced symptom will recur (0 *not likely at all* to 5 *extremely likely*) and the fear that it will recur (0 *not worried at all* to 5 *extremely worried*). The symptom with the highest sum score was selected as the topic for the task. We framed the participants with an approximate maximum search time of 15 minutes. No further guidance or instructions on what or how to search were provided. The exact task text can be found in Textbox 1. Desktop activity was recorded using Open Broadcaster Software [63]. After the task, the participants received another questionnaire regarding their search, including items on ecological validity, search appraisal, and their stress level.

**Task description.**

Imagine that the symptom ______ is acute or recently in the past. You now have a maximum of 15 minutes to search for information on the internet. Search like you would at home. There is no right or wrong approach in doing this. When you think you are done, report to the experimenter.

### Ethics Approval

Ethical approval for this study was obtained from the institutional review board of the University of Klagenfurt on April 2, 2019 (2018-116).

### Transcribing of Video Data

The software application ELAN [64] was used to determine the time spans and number of clicks for each recorded search session (ie, search duration, total clicks, unique resources, search queries, and page views). In addition, 3 undergraduate psychology students independently transcribed the videos. Intraclass correlation coefficients (ICCs; *k*=3, absolute-agreement, 2-way mixed-effects model) were excellent for total clicks (ICC=0.99), unique resources (ICC=0.99), search queries (ICC=0.99), page views (ICC=0.99) and search duration (ICC=1.00). If there was complete agreement among all 3 raters, the respective value was used. In case of agreement between at least two raters, this value was used. For discordance in all 3 raters, the median was used.

### Measures

#### Demographics and General Questions on OHIS

Data on age, gender, citizenship, occupation, education, and civil status were collected as part of the sociodemographic characteristics. Moreover, we assessed relationship status, the length of the relationship in years, the age of the youngest child in months, and the total number of children in the household. Items on OHIS behavior, in particular, included the weekly time spent, the number of days during the week, the average time spent for an individual search session, the used device, and for whom the searches are (ie, self, child, partner, relative, friends, and others).

#### eHealth Impact Questionnaire

The eHealth Impact Questionnaire (Part 1) by Kelly et al [65] is an 11-item scale for measuring the attitude to use the web for health-related purposes. The scale is divided into two subscales with 5 items on attitudes toward web-based health information and 6 items regarding attitudes toward sharing health experiences on the web. Part 2 of this scale was not included because that measure is for the evaluation of single websites that do not match with this study design. Each item is scored on a 5-point Likert scale ranging from 1 (strongly disagree) to 5 (strongly agree). Scores were calculated by transforming the raw scores into a metric ranging from 0 to 100. The internal consistency in this sample was Cronbach α=.67 for the first subscale and Cronbach α=.71 for the second subscale.

#### German eHealth Literacy Scale

The German eHealth Literacy Scale by Soellner et al [66] is the translated version of the eHealth Literacy Scale by Norman and Skinner [67] for assessing self-perceived health problem-solving skills in electronic environments. The scale consists of 8 items rated on a 5-point Likert scale. In our sample, the German eHealth Literacy Scale demonstrated acceptable internal consistency, with Cronbach α=.78.

#### PHQ-15 Measures

The PHQ-15 by Kroenke et al [68] is an instrument for the screening of somatic symptoms and the severity of somatization. The inventory contains 15 items on different somatic symptom groups, which cover more than 90% of the presented symptoms in primary care. In contrast to the original version, we asked only in a binary response format (yes or no) about the occurrence within the last 2 weeks to keep the scenario topic as current as possible. The following symptoms were removed for the by-proxy group because they were not appropriate for young children or commonly reported health symptoms: menstrual cramps, pain or problem during sexual intercourse, chest pain, dizziness, and having low energy. We replaced them with skin rash, fever, earache, vomiting, cough, sore throat, and uncontrollable crying. For both groups, an option was provided to write in symptoms not listed.

#### Modified Short Health Anxiety Inventory

The Modified Short Health Anxiety Inventory (mSHAI) by Bailer et al [69] is a 14-item scale for assessing health anxiety. Initially published by Salkovskis et al [70] as the Short Health Anxiety Inventory, the mSHAI has, in contrast, an abridged response format and is proposed as unidimensional. On the basis of the previous 6 months, participants rate their fear of illness on a 5-point Likert scale, with higher values indicating greater fear of illness. Total scores range between 0 and 56. The health anxiety inventory was highly reliable in this sample, with a Cronbach α of .91.

#### mSHAI-Child

The mSHAI-Child is an adaption of the mSHAI by Bailer et al [69] for the self-report measurement of health anxiety by proxy toward the child. A similar approach was conducted with health anxiety by proxy in pregnant mothers [71]. Parents rate their health-related anxiety regarding their youngest child on a scale of 0 (strong disagreement) to 4 (strong agreement) on each of the 14 items, with a total score range of 0 to 56. In this study, the Cronbach α was good (α=.89).

#### State Anxiety

The Short State Anxiety Inventory by Grimm [72] is a 10-item scale to measure current emotional stress and anxiety. The German translation was published by Laux et al [73] and is originally based on the State-Trait Anxiety Inventory [74]. Participants rate on an 8-point Likert scale their tension, nervousness, and apprehension. The higher the total score, the higher the level of state anxiety and stress. Participants completed the questionnaire twice, immediately before and after the search task. In this study, the internal consistencies were Cronbach α=.66 at the preassessment stage and Cronbach α=.74 at the postassessment stage.

#### Couple Satisfaction Index

The Couple Satisfaction Inventory–32 by Funk and Rogge [75] is a global self-report measurement of satisfaction in a relationship. The 32-item version of the Couple Satisfaction Inventory is psychometrically sound and precise for detecting differences in the level of relationship satisfaction. Total scores can range between 0 and 161, with higher scores indicating better couple satisfaction. Funk and Rogge [75] recommended a distress cut-off of 104.5 to identify distressed relationships. In this study, the Cronbach α was excellent (α*=*.96).

#### Parenting Stress Scale

The Parental Stress Scale by Berry and Jones [76] is an 18-item measure for quantifying stress that results from the parent-child relationship. The scale covers different components of stress during parenthood, including parental rewards, parental stressors, parental satisfaction, and lack of control. Total scores range from 18 to 90, with higher scores indicating greater parental stress. The Parental Stress Scale showed acceptable internal consistency in our sample (Cronbach α=.73).

#### Subjective Search Evaluation

We developed 6 items for the evaluation of search appraisal and the impact of participants’ web-based search on their behavior based on a review of previous literature and pilot testing [20]. Scoring ranged from 0 (strong disagreement) to 4 (strong agreement) for each item. This included (1) search satisfaction with the progress of the search (“I am satisfied with the way my search has gone”), (2) search success of the final result of the search (“I am satisfied with the result of my search”), (3) self-empowerment that originates from the search (“The search makes me feel more self-empowered than before”), (4) the presence of an information overload during the task (“There was a point during the search when I felt overwhelmed by the amount of information”), (5) the need to contact a physician and discuss the information with him or her (“I will discuss the information found with my doctor”), and (6) the presence of unmet information needs (“I now have more open questions than before”).

#### Objective Search Characteristics

Quantitative search characteristics were extracted from the recorded desktop activity. These included the duration of the search, from the start of the first keystroke to the last significant mouse movement (*search duration*); the sum of all clicks that lead to visible actions (*total clicks*); the number of performed search queries (*search queries*); the number of unique accessed webpages, including search engine result pages, websites, and their subpages (*page impressions*); and the number of resources used during the search (*unique resources*).

## Results

### General OHIS Behavior

Parents report seeking on the web for health information for their child (44/46, 96%) rather than for themselves (37/46, 80%), followed by searching for their intimate partner (19/46, 41%), relatives (10/46, 22%), and friends (7/46, 15%). Most parents spend up to 1 hour weekly on OHIS (32/46, 70%). The average time for search session at home varied greatly in the sample: 1 to 5 minutes (11/46, 24%), 5 to 10 minutes (13/46, 28%), 10 to 20 minutes (12/46, 26%), 20 to 40 minutes (5/46, 11%), 40 to 60 minutes (4/46, 9%), and no answer (1/46, 2%).

### Topic of the Search Task

Across groups, searches were carried out for 17 different symptoms. The most common scenario in the self-seeker group was back pain (5/23, 22%), whereas the most common topic in the by-proxy group was cough (8/23, 35%). A total of 2 participants searched for own suggested topic (ie, eye inflammation and common cold). A minority had already searched the internet for the respective symptom in the past 4 weeks (10/46, 22%). Less than half of the sample (20/46, 43%) had already seen their physician about the symptom. All topics of the search task are listed in Table 2.

### Analysis of Search Queries

Parents performed on average 2.95 search queries (SD 1.83) during their search. On the basis of the first search query, most parents started the search on symptoms (40/46, 87%); however, a small number of participants initially looked for treatments (4/46, 9%) or specific diseases (2/46, 4%). Of the 46 parents, 44 (96%) used Google as the search engine. In addition, of the 46 parents, only 1 (2%) started with an alternate search engine, whereas 1 (2%) began to seek in Facebook groups. A minority began with a single keyword search (7/45, 16%), whereas most participants used key phrases of >1 keyword (38/45, 84%). The 22 parents who searched for their child (mean 3.77, SD 1.75) compared with the 23 participants who searched for themselves (mean 2.43, SD 1.27) used significantly more words in their initial search term (t_43_=−2.86, *P*=.01). Many parents in the by-proxy group specified their search terms with the child’s age to find more suitable results. In the overall sample, the average position of the organic search result clicked first was 2.40 (SD 3.19), suggesting high attention on the top search results. Nearly all parents (40/46, 87%) stayed on the first page of the search engine results and never clicked on page 2 or further. During the search sessions, only 4% (2/46) of the parents clicked on an advertisement within the Google search results.

**Table 2:** Scenario topic.

Group and scenario		Times searched, n
**Self-seeker group (n=23)**		
	Back pain	5
	Headaches	4
	Feeling tired or lack of energy	3
	Vertigo	3
	Diarrhea	2
	Elbow pain	1
	Stomachache	1
	Trouble sleeping	1
	Menstrual pain	1
	Tachycardia	1
	Eye inflammation	1
**By-proxy-seeker group** **(n=23)**		
	Cough	8
	Stomachache	3
	Diarrhea	3
	Headaches	2
	Fever	2
	Skin rash	2
	Sore throat	1
	Nausea	1
	Common cold	1

### Correlations Among Study Variables

Multimedia Appendix 1 shows the correlations among all study variables. Health anxiety was moderately negatively associated with search satisfaction (*r*_s_=−0.34, *P*=.02) and search success (*r*_s_=−0.39, *P*=.01) and moderately positively associated with the need to talk to a physician after the search (*r*_s_=0.31, *P*=.03) and unmet information needs (*r*_s_=0.30, *P*=.04). eHealth literacy was positively correlated with the attitude toward web-based health information (*r*_s_=0.35, *P*=.01) and search satisfaction (*r*_s_=0.50, *P*<.001), whereas a moderate negative correlation was observed with unmet information needs (*r*_s_=−0.32, *P*=.02) and information overload (*r*_s_=−0.30, *P*=.04). No associations were found between relational variables (ie, couple satisfaction and parental stress) and any search-related variables. Perceived stress after the search was negatively correlated with search success (*r*_s_=−0.33, *P*=.02) and positively correlated with the need to talk to a physician (r_s_=0.31, *P*=.03); however, this was not the case for other evaluation items or characteristics of the search. In general, our analysis showed few associations between the chosen inventories (ie, health anxiety, eHealth literacy, attitudes toward web-based information, and stress) and objective search characteristics. Similarly, there was only 1 significant association between the items on search evaluation and objective search characteristics, suggesting the absence of a clear relationship between appraisals and the manner of searching the web for symptoms.

### Comparison of Self-seeker and By-proxy Seeker

A series of independent sample 2-tailed *t* tests were conducted between both experimental conditions (Table 3). As expected, due to the randomization, no significant differences were found for in baseline variables. There were also no significant differences in any of the 6 items on the evaluation of the health-related search. Parents who searched for their child reported a greater need to communicate with a physician than those who searched for themselves, but this difference was not statistically significant with the current sample (*U*=187.5, *z*=−1.78, *P*=.07), although the effect size was moderate (Cohen *d*=.58).

**Table 3:** Descriptive statistics and 2-tailed *t* tests or Mann-Whitney U tests for the comparison of self-seeker and by-proxy seeker^a^.

Measure		Self-seeker, mean (SD)	By-proxy seeker, mean (SD)	2-tailed *t* test (*df*)	*Z*	*P* value
mSHAI^b^		11.60 (8.70)	12.65 (10.25)	−0.37 (44)	N/A^c^	.71
mSHAI-Child^d^		14.82 (9.48)	16.04 (9.37)	−0.43 (44)	N/A	.66
G-eHEALS^e^		30.21 (3.84)	30.86 (3.74)	−0.58 (44)	N/A	.56
eHIQ-Ohis^f^		55.86 (18.80)	62.39 (17.76)	−1.20 (44)	N/A	.23
eHIQ-Share^g^		70.65 (16.20)	69.56 (13.90)	0.24 (44)	N/A	.80
CSI-32^h^		134.70 (15.84)	130.68 (24.19)	0.61 (37)	N/A	.54
PSS^i^		34.68 (7.27)	35.69 (6.10)	−0.50 (43)	N/A	.61
Stress before the task^j^		24.43 (7.56)	27.30 (10.26)	−1.07 (44)	N/A	.28
Stress after the task^k^		23.60 (10.27)	28.00 (9.56)	−1.50 (44)	N/A	.14
**Objective search characteristics**						
	Search duration^l^	562.95 (338.22)	525.08 (302.90)	0.40 (44)	N/A	.69
	Total clicks^m^	27.56 (25.61)	21.26 (14.60)	1.02 (44)	N/A	.31
	Page impressions^n^	9.56 (5.46)	9.82 (5.79)	−0.15 (44)	N/A	.87
	Unique resources^o^	5.17 (4.27)	4.69 (2.77)	0.45 (44)	N/A	.65
	Search queries^p^	2.60 (1.55)	3.30 (2.05)	−1.29 (44)	N/A	.20
**Subjective search evaluation**						
	Search satisfaction^q^	2.96 (0.82)	3.30 (0.63)	N/A	−1.45	.14
	Search success^r^	3.30 (0.87)	3.48 (0.73)	N/A	−0.60	.54
	Self-empowerment^s^	1.83 (1.11)	2.22 (1.08)	N/A	−1.17	.23
	Information overload^t^	1.22 (1.31)	0.91 (0.99)	N/A	−0.53	.59
	Need to talk to a physician^u^	0.83 (1.02)	1.52 (1.31)	N/A	−1.78	.07
	Unmet seeking needs^v^	0.74 (0.86)	0.83 (0.83)	N/A	−0.49	.62

^a^Independent sample 2-tailed *t* tests for inventories and objective search characteristics. Mann-Whitney *U*-test for subjective search evaluation.

^b^mSHAI: Modified Short Health Anxiety Inventory.

^c^N/A: not applicable.

^d^mSHAI-Child: Modified Short Health Anxiety Inventory (by proxy related to own child).

^e^G-eHEALS: German eHealth Literacy Scale.

^f^eHIQ-Ohis: eHealth Impact Questionnaire, attitudes toward web-based health information.

^g^eHIQ-Share: eHealth Impact Questionnaire, attitudes toward sharing health experiences.

^h^CSI-32: Couple Satisfaction Index-32.

^i^PSS: Parental Stress Scale.

^j^Stress before the task: measured with the Short State Anxiety Inventory.

^k^Stress after the task: measured with the Short State Anxiety Inventory.

^l^Search duration: length of the search session (in seconds).

^m^Total clicks: the sum of all clicks during the search session that lead to visible actions.

^n^Page impressions: number of unique accessed webpages during the search session.

^o^Unique resources: number of resources used during the search session.

^p^Search queries: number of performed search queries.

^q^Search satisfaction: “I am satisfied with the way my search has gone.”

^r^Search success: “I am satisfied with the result of my search.”

^s^Self-empowerment: “The search makes me feel more self-empowered than before.”

^t^Information overload: “There was a point during the search when I felt overwhelmed by the amount of information.”

^u^Need to talk to a physician: “I will discuss the information found with my doctor.”

^v^Unmet seeking needs: “I now have more open questions than before.”

For self-seekers, the results from the pretest (mean 24.43, SD 7.56) and posttest (mean 23.60, SD 10.27) stress indicate that the search task did not result in an increase of stress (paired t_44_=0.43, *P*=.66). There was also no significant increase for the by-proxy seeker group in stress before the search task (mean 27.30, SD 10.26) compared with that after the search task (mean 28.00, SD 9.56; paired t_44_=−0.34, *P*=.73). Moreover, no differences were found for total clicks, page impressions, number of unique resources, and search queries (*P*>.05).

## Discussion

### Principal Findings

This study investigated the relationship between health anxiety, eHealth literacy, search appraisals, and quantitative search characteristics in the context of a symptom-driven web search. Few differences between self-seekers and proxy-seekers were found, but there was trend for parents searching for their young child to report a higher need to communicate with a physician than those searching for themselves. Consistent with previous experimental studies, this study contributes further evidence to the importance of trait health anxiety. In contrast to Singh and Brown [55] or Jungmann et al [57], we focused on the comparison of self-seekers and proxy seekers as well as the connection with search appraisals, thus expanding the contemporary understanding of OHIS processes and their evaluation by consumers. Contrary to expectations based on the literature, eHealth literacy was related neither to search success nor to a more efficient search.

### Comparison With Previous Work

Not surprisingly, almost all parents used the Google search engine as the first entry point, which is in full agreement with other studies [20,56]. We observed that the entire symptom search experience primarily occurred on the first search result page within the top rankings. Similarly, Beus [77] has also found a click rate of more than 50% for the first 3 organic search results in mobile searches. Google’s ranking algorithm is important in influencing how fast consumers find health information. In our experiment, parents specified their search query term rather than searching more in depth when results were not perceived as useful. In general, the search queries parents used on behalf of their children tended to be more elaborate, for example, with precise age information. This observation has implications for providers of pediatric health content on the web. For pediatric health information, age-specific information in combination with specific symptoms may be most helpful in meeting parents’ needs. Given the fact that uncertainty is a potential difficulty during OHIS [78,79] and symptoms have different health implications based on developmental age, this approach could support parents’ connection to information that is more developmentally appropriate for their child.

Furthermore, advertisements both in Google search results and on further websites received little to no attention. This could be due to a general blindness for web-based advertisements [80] as well as the mistrust that consumers attribute to advertisements on the internet [81-83]. This is an important finding for possible future interventions as it suggests that target group-oriented advertising may not work in symptom-related searches, for example, to address stressed parents with relevant information directly during their search. Previous research has also shown that health websites with advertisements are perceived as less trustworthy [84]. Therefore, it could be more promising to provide parents with relevant eHealth knowledge via social media influencers, as there may be a pre-existing higher level of trust [85] that is lacking in conventional text advertising.

Similar to previous findings, we found a relationship between trait health anxiety and the need to talk to a physician after the search [37,57] and poorer search outcomes [38]. The relationship between pretask stress and health anxiety was also significant but not that between health anxiety and change in stress after the task. The experimental setting may have increased the baseline levels of pretask stress. Although not reported in this study, physiological data were also collected and showed a downward trend of electrodermal activity in many participants, which may have made detecting individual differences in change in stress after the task more difficult.

There was no association of eHealth literacy with search success, self-empowerment, need to talk to a physician, search duration, number of search queries, and stress after the search. We hypothesized that parents with higher eHealth literacy would search the web faster, use fewer resources, need fewer search queries, and thus search more efficiently. In theory, individuals with higher eHealth literacy should be better at finding, selecting, and using health information on the web than individuals with lower eHealth literacy. The nonsignificant results may raise concerns about the general difficulty of valid assessment of eHealth literacy using previous methods. The validity problems of the eHealth Literacy Scale have been described in the literature [86,87] and are generally attributable to the self-assessment character of the scale [87,88]. Parents with low eHealth literacy may not be aware of their lack of competence, whereas parents with high eHealth literacy may underestimate their skills [89,90]. In a study by Meppelink et al [91] on vaccination information seeking on the web, confirmation bias was more prevalent in parents with high health literacy. Prospectively, the dissemination on pitfalls and common cognitive biases of OHIS [92] could be valuable for enhancing eHealth literacy and mitigating the ramifications of maladaptive OHIS. A review by Karnoe and Kayser [93] found that although there are alternative methods of measuring eHealth literacy, these need further testing for use in research.

Contrary to expectations, we found no statistically differences between parents who searched for themselves and those who searched for their child, although these results should be interpreted tentatively due to the small sample size. We did find a trend that parents who searched for their child reported a higher need to talk to a physician after the search than among parents who searched for themselves (*P*=.07, Cohen *d*=.58). This finding should be explored further in a large and more diverse sample of parents. Regarding other differences, we had hypothesized that a higher level of self-perceived responsibility and uncertainty during managing children’s symptoms in by-proxy seekers would lead to poorer outcomes in various dimensions (ie, search appraisals, longer search time, and higher stress). Although previous research has shown substantial differences between the characteristics of self-seekers and by-proxy seekers [18,94-96], these studies did focus neither on intraindividual differences on web users who usually act in both roles (ie, parents) nor on search appraisals. Nevertheless, we have several explanations for these unexpected results. First, there is the absence of considerable differences in most of the dimensions examined between self-seekers and by-proxy seekers exclusively in parents. In contrast to previous studies [18,94-96], our sample consisted only of parents. Within the general research on OHIS by proxy, the parent-child relationship may differ from OHIS on behalf of intimate partners, elderly relatives, informal caregivers, or friends. Parents are responsible for their children’s health, whereas in most other by-proxy search relationships, there is still a certain degree of personal responsibility. Reifegerste and Bachl [97] found that relationship closeness was a relevant factor for OHIS by proxy, and that motives can differ across various by-proxy search types (ie, between parent-child and parent-partner). Thus, parents’ OHIS for themselves could be very similar to OHIS by proxy for their children but may differ considerably for other relationships. Second, although the symptoms were derived from the recent past, they may no longer be relevant at the time when participants processed the task in the laboratory. Acuteness, perceived information need, and risk perception are important catalysts for OHIS [98-100]. Additional findings from Rains and Tukachinsky [101] suggest the association between information seeking depth on the web (ie, number of webpages) and the uncertainty appraisal intensity. Future research in larger samples and natural settings is essential, for example, with the support of smartphone apps that record health-related symptom searches just in time.

Finally, a recent scoping review revealed a wide variety of information needs among internet users [102], suggesting the requirement for better-tailored web-based health resources at the individual level. In addition to frequently examined relating factors on search success, such as credibility, trust, or information quality [103,104], other factors should also increasingly be included in theoretical considerations and tested experimentally, for example, contemporary features of social media and their effectiveness on knowledge transfer [105,106]. Likewise, the use of artificial intelligence-based chatbots could be very promising for addressing the individual needs of consumers during OHIS [107-109]. Although in our experiment we only assessed subjective search success and search satisfaction, future studies could evaluate the respective website elements of a health-related website more precisely in terms of their contribution to search success. Approaches that consider perceived user trust and enhance interactivity may significantly improve users’ experience with health websites [110].

### Limitations

Our work has some limitations. The experiment was based on a small sample size and did not allow any conclusions about the search behavior of the public. The sample consisted mainly of mothers with a high level of education and therefore underrepresented both fathers and parents with lower educational attainments. Neither the medical knowledge nor the possible work in a medical profession was assessed. Previous expertise could have had a substantial impact on the search and its evaluation. Furthermore, we applied an experimental approach in a laboratory setting; thus, the findings might not be generalized to real search behavior at home, although parents reported that their search behaviors were similar to those at home. In addition, we used single items for evaluating the subjective search outcomes and thus may have overlooked important dimensions. Future studies should apply more sophisticated assessment approaches in this context. Finally, the lack of association between eHealth literacy and search characteristics in our study may be related to the type and complexity of the search task. Further studies should examine the relationship between eHealth literacy and search characteristics based on the perceived task difficulty.

### Conclusions

The results of this study indicate that parents’ symptom search evaluation is considerably associated with health anxiety, less with eHealth literacy, and not significantly with attitudes toward OHIS. These findings contribute additional evidence to a growing body of literature on the role of health anxiety during OHIS for oneself and others. Given the prevalence of web-based health information use among parents, further research is urgently needed to provide evidence-based recommendations on how to search the web most effectively and how this connects with subsequent health behaviors.

## Appendix

Multimedia Appendix 1 Spearman correlations for inventories, search evaluation items, and search characteristics.

## Abbreviations

ICC: intraclass correlation coefficient
mSHAI: Modified Short Health Anxiety Inventory
OHIS: online health information seeking
PHQ-15: Patient Health Questionnaire-15
