# Supplementary material for: Online Health Information Seeking for Self and Child: An Experimental Study of Parental Symptom Search
Source: JMIR Pediatr Parent. 2022 May 9;5(2):e29618. doi: 10.2196/29618 (PMC9127650; doi:10.2196/29618)
Supplement: Multimedia Appendix 1 [file pediatrics_v5i2e29618_app1.pdf]

Multimedia Appendix 1. Spearman correlations for inventories, search evaluation items, and search characteristics.[a]

Variable	1	2	3	4	5	6	7	8	9	10	11	12	13	14	15	16	17	18	19	20
1. mSHAI[b]	—[c]																			
2. mSHAI-Child[d]	0.58[e]	—																		
3. G-eHEALS[f]	−0.13	−0.06	—																	
4. eHIQ-Ohis[g]	0.27	0.03	0.35[h]	—																
5. eHIQ-Share[i]	0.10	0.07	0.19	0.34[h]	—															
6. CSI-32[j]	0.00	0.06	0.11	−0.09	0.00	—														
7. PSS[k]	0.22	0.17	0.12	0.42[e]	−0.07	−0.38[h]	—													
8. Stress before the task[l]	0.37[e]	0.23	−0.06	0.22	−0.05	−0.04	0.61[e]	—												
9. Stress after the task[m]	0.23	0.24	−0.01	0.17	−0.01	−0.13	0.33[h]	0.55[e]	—			.								
10. Search satisfaction[n]	−0.34[h]	−0.04	0.50[e]	0.22	0.13	0.02	−0.04	−0.16	−0.22	—										
11. Search success[o]	−0.39[h]	0.01	0.22	0.00	−0.03	0.05	−0.08	−0.35[h]	−0.33[h]	0.60[e]	—	.								
12. Self empowerment[p]	−0.11	−0.01	0.24	0.27	0.00	0.09	0.03	−0.08	−0.13	0.40[e]	0.46[e]	—								
13. Information overload[q]	0.11	−0.04	−0.30[h]	−0.00	−0.13	−0.30	0.08	0.13	0.25	−0.37[e]	−0.37[e]	−0.06	—							
14. Need to talk to a physician[r]	0.31[h]	0.30[h]	−0.07	−0.03	−0.04	0.13	−0.00	0.13	0.31[h]	−0.02	−0.15	0.24	0.19	—						
15. Unmet seeking needs[s]	0.30[h]	0.06	−0.32[h]	0.00	−0.17	−0.02	0.21	0.28	0.19	−0.48[e]	−0.50[e]	−0.33[h]	0.47[e]	0.16	—					
16. Search duration[t]	0.01	−0.18	0.08	0.13	0.08	0.22	−0.05	−0.05	−0.10	0.11	−0.18	0.20	−0.13	0.14	0.09	—				
17. Total clicks[u]	0.00	−0.09	0.14	0.06	−0.10	0.06	0.01	0.02	0.09	−0.01	−0.26	−0.00	0.08	0.07	0.12	0.64[e]	—			
18. Page impressions[v]	0.02	−0.15	0.22	0.25	−0.05	0.01	0.07	−0.01	0.03	0.01	−0.17	0.04	0.03	−0.02	0.10	0.63[e]	0.84[e]	—		
19. Unique resources[w]	0.13	−0.01	0.03	0.11	−0.05	0.02	0.01	.04	0.07	−0.00	−0.18	−0.03	0.10	0.07	0.23	0.60[e]	0.70[e]	0.80[e]	—	
20. Search queries[x]	−0.06	−0.25	0.20	0.31[h]	−0.09	−0.04	0.19	0.09	0.07	0.04	−0.29[h]	−0.07	0.03	−0.04	0.15	0.40[e]	0.47[e]	0.63[e]	0.37[h]	—

[a]N=46, with the exception of CSI-32 (n=39) and PSS (n=45).

[b]mSHAI: Modified Short Health Anxiety Inventory.

[c]Not applicable.

[d]mSHAI-Child: Modified Short Health Anxiety Inventory (by proxy related to own child).

[e]*P*<.01.

[f]G-eHEALS: German eHealth Literacy Scale.

[g]eHIQ-Ohis: eHealth Impact Questionnaire, attitudes toward web-based health information.

[h]*P*<.05.

[i]eHIQ-Share: eHealth Impact Questionnaire, attitudes toward sharing health experiences.

[j]CSI-32: Couple Satisfaction Index–32.

[k]PSS: Parental Stress Scale.

[l]Stress before the task: measured with the Short State Anxiety Inventory.

[m]Stress after the task: measured with the Short State Anxiety Inventory.

[n]Search satisfaction: "I am satisfied with the way my search has gone."

[o]Search success: "I am satisfied with the result of my search."

[p]Self-empowerment: "The search makes me feel more self-empowered than before."

[q]Information overload: "There was a point during the search when I felt overwhelmed by the amount of information."

[r]Need to talk to a physician: "I will discuss the information found with my doctor."

[s]Unmet seeking needs: "I now have more open questions than before."

[t]Search duration: length of the search session (seconds).

[u]Total clicks: the sum of all clicks during the search session that lead to visible actions.

[v]Page impressions: number of unique accessed webpages during the search session.

[w]Unique resources: number of resources used during the search session.

[x]Search queries: number of performed search queries.
